# Quantitative investigation of cardiac motion effects on in vivo diffusion tensor parameters: a simulation study

**DOI:** 10.1186/1532-429X-15-S1-P244

**Published:** 2013-01-30

**Authors:** Hongjiang Wei, Magalie Viallon, Benedicte M Delattre, Lihui Wang, Vinay M Pai, Han Wen, Hui Xue, Christoph Guetter, Marie-Pierre Jolly, Pierre Croisille, Yuemin Zhu

**Affiliations:** 1 Creatis; CNRS (UMR 5220), Inserm (U1044); Insa Lyon, Villeurbanne, France; 2 Department of Radiology, University Hospitals of Geneva, Geneva, Switzerland; 3 BBC/NHLBI/NIH, Bethesda, MD, USA; 4 Siemens Corporate Research, Princeton, NJ, USA; 5 Jean-Monnet University, Saint-Etienne, France

## Background

Cardiac motion is a crucial problem in in vivo diffusion tensor imaging (DTI) of the human heart. Despite its importance, the effects of cardiac motion on diffusion tensor parameters of the human heart in vivo have not been well established, mainly because of large signal loss. Recently, an efficient method was proposed that acquires cardiac diffusion weighted (DW) images at different time points of the cardiac cycle and reduces motion-induced signal loss using PCA filtering and temporal MIP techniques (PCATMIP) (Rapacchi, Invest Radiol 2011). Meanwhile, polarized light imaging (PLI) provides us the ground-truth of the heart fiber architecture, and DENSE sequence offers us higher spatial resolution displacement fields of the human heart in vivo. These different imaging possibilities have led us to investigate a multimodal approach to quantitatively investigate the effects of cardiac motion on diffusion tensor parameters such as fractional anisotropy (FA), mean diffusivity (MD) and fiber angles.

## Methods

**Figure 1:**
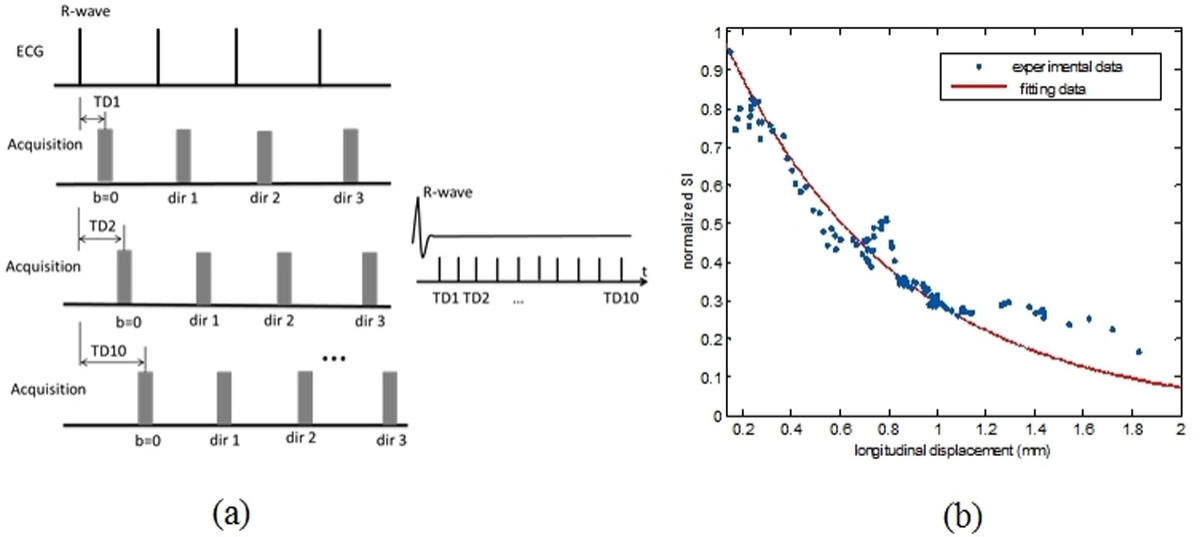
(a) The acquisition scheme used in DWI. The TD of the DW images was determined by DENSE acquisition. (b) Normalized myocardium DW signal intensity as a function of the mean cardiac motion. The cardiac motion is represented as the relative longitudinal displacement amplitude between two continuous time points of DENSE acquisition.

The PLI data was acquired on an ex vivo heart using the imaging technique described in (Jouk PS, Eur J Cardiothoracic Surg 2007) and the in vivo DTI experiments was performed on a 1.5T scanner involving 6 volunteers. The method consists of using Monte-Carlo simulation to generate realistic DW images from PLI, obtaining motion information from DENSE acquisition and DW images at the same trigger delay (TD) (acquisition scheme in Fig. 1(a)), constructing an empirical model to describe the relation between motion and diffusion signal intensity (Fig. 1(b)), applying such model to the original simulated DW images in order to obtain the motion-induced datasets, and applying the PCATMIP technique to simulated data for obtaining the motion-reduced DW images.

## Results

**Figure 2:**
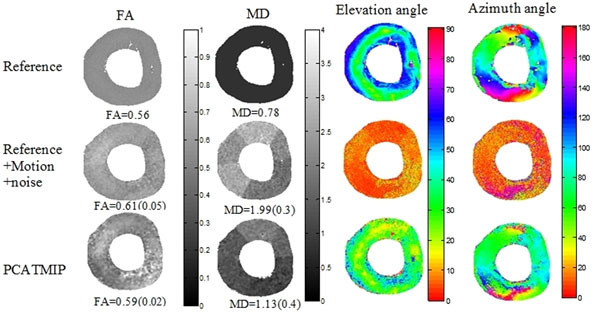
Impacts of cardiac motion on FA, MD and fiber orientation.

Cardiac motion induced an overestimation of FA and MD and a reduced range of HA (Fig. 2). After processing by PCATMIP, both FA (0.59 ± 0.02) and MD (1.13 ± 0.4 × 10-3mm2/s) are smaller than those obtained from motion-induced acquisition (0.61±0.05 and 1.99 ± 0.3 × 10-3 mm2/s, respectively). The regular variation pattern of elevation and azimuth angles is missing after adding the cardiac motion and Rician noise. The signal loss due to the motion and noise therefore greatly influences the angle maps. After using PCATMIP method, such regular azimuth angle variation patterns were nearly recovered despite a relative higher noise level. The elevation angle range was from 41 ± 13 degree on the endocardium and back to 35 ± 12 degree on the epicardium for the left ventricle, which reflects the fiber rotation.

## Conclusions

This study demonstrates that cardiac motion introduces an overestimation for FA and MD. Using the proposed motion model and the PCATMIP method, measurement accuracy on diffusion tensor parameters was significantly improved, which suggests new solutions to the problem of getting insights into in vivo fiber architecture of the human heart.

## Funding

This work was supported by the French ANR 2009 (under ANR-09-BLAN-0372-01).

